# Editorial: Community reintegration after spinal cord injury

**DOI:** 10.3389/fresc.2022.1020279

**Published:** 2022-09-20

**Authors:** Kristin E. Musselman, Christina Papadimitriou, Elena Vasilchenko

**Affiliations:** ^1^ Department of Physical Therapy and Rehabilitation Sciences Institute, University of Toronto, Toronto, ON, Canada; ^2^ SCI Mobility Lab, KITE, Toronto Rehabilitation Institute-University Health Network, Toronto, ON, Canada; ^3^ Department of Interdisciplinary Health Sciences, Oakland University, Rochester, MI, United States; ^4^ FSBI “Novokuznetsk Scientific and Practical Centre for Medical and Social Expertise and Rehabilitation of Disabled Persons” of the Ministry of Labour and Social Protection of the Russian Federation, Novokuznetsk, Russia

**Keywords:** spinal cord injury, community integration, participation, rehabilitation, inclusion

**Editorial on the Research Topic** Community reintegration after spinal cord injury by Musselman KE, Papadimitriou C, Vasilchenko E. (2022) Front. Rehabil. Sci. 3:1020279, doi: 10.3389/fresc.2022.1020279

Sustaining a spinal cord injury (SCI) is a catastrophic event that approximately 250,000–500,000 people experience every year (1). In addition to the sensory, motor and autonomic dysfunction caused by damage to the spinal cord, people living with SCI often experience debilitating secondary complications, such as pressure injuries and chronic pain. Physical rehabilitation following injury often results in improved motor task performance, such as greater skill and independence in transferring, walking and wheeling (2). However, over the past several decades the rehabilitation paradigm has shifted to a biopsychosocial model in which the medical, psychological and social aspects of the injury are simultaneously addressed (3). As a result, community integration is now a core goal of rehabilitation.

As rehabilitation professionals and researchers, we often expect improvements in function to translate to community integration and inclusion. Integration into the community is a multi-faceted issue that refers to participation in society, including engagement in occupation, recreation, social activities and relationships with others. Related to the concept of community integration is inclusion, which is the practice of creating environments and situations where all people have equal access to opportunities, resources and knowledge regardless of their abilities. Although community integration is a key goal of SCI rehabilitation, it is recognized as a challenge to obtain through rehabilitation (4). A recent longitudinal study demonstrated that within the first year following SCI, 55% of the participants struggled with social participation, defined as “the degree to which a person integrates and interacts in their community”, and only one third were employed (5).

As the lengths of hospital stays and inpatient rehabilitation after SCI are decreasing in Canada and the United States (6, 7), there is less time to help people with SCI prepare for community integration. Yet, the quantity of inpatient rehabilitation services received is only weakly associated with participation one year post-SCI (6). Hence, consideration of alternative settings and methods for community integration programs is warranted. As there is a pressing need to increase our understanding of the factors that affect successful integration and inclusion, and to develop strategies to facilitate meaningful participation, we created a Research Topic to highlight current knowledge and innovations in the area of community integration and inclusion following SCI.

The Research Topic consists of four original articles sharing research that is not only innovative, but also responsive to the rapidly changing health care environment of the past few years. The COVID-19 pandemic and the “emerging public health crisis” of falls (8), are two issues studied. Both pandemic restrictions and a fear of falling may be associated with significant physical and social isolation, which affect one's ability to function in the community. Simpson et al. (2022) explored the impact of the first wave of the COVID-19 pandemic on the participation, mobility and well-being of community-dwelling individuals with SCI. The findings highlight the pervasiveness of ableism in our health care environments Simpson et al. (2022). Chan et al. (2022) examined the impact falling and living with a fear of falling have on individuals within the first year of SCI. Krysa et al. (2022) explored the rehabilitation experiences of people living with SCI in relation to the transition between hospital and community. Both strengths and shortcomings of the rehabilitation journey were identified, highlighting opportunities to better support transitions in care for those living with SCI; for example, earlier education on self-management, greater opportunities to learn about community supports while in the hospital and more empathetic communication from health care professionals Krysa et al. (2022). The need to extend rehabilitation services beyond inpatient rehabilitation motivated the study by Rimmer and colleagues Rimmer et al. (2022), which focused on the perceived outcomes of a community-based, virtual wellness intervention for individuals living with SCI. Understanding the acceptance and perceived value of virtual interventions is timely, but also addresses a health care need identified by the SCI population long before the COVID-19 pandemic Simpson et al. (2022).

A common thread in these four studies is the collection and reporting of the perspectives of people living with SCI, whether through self-report questionnaires Chan et al. (2022) or semi-structured interviews (Simpson et al. (2022), Krysa et al. (2022), Rimmer et al. (2022)). Understanding an issue or phenomenon from the perspectives of those with lived experience is a catalyst for the development of evidence-based and person-centered solutions to health care challenges (9). As demonstrated in this Research Topic collection, people living with SCI desire person-centered rehabilitation throughout the continuum of care. Moreover, study participants highlighted factors and strategies that may facilitate participation, inclusion and community integration. For example, after sustaining a SCI it is important to foster resiliency Simpson et al. (2022), empowerment Krysa et al. (2022) and peer support Rimmer et al. (2022), along with hope for the future. This knowledge may help us break down barriers to community integration and create inclusive health care services for people living with SCI.

## References

[1] World Health Organization. Spinal cord injury (November 19, 2013). Available at: https://www.who.int/news-room/fact-sheets/detail/spinal-cord-injury (cited August 15, 2022).

[2] Harvey LA. Physiotherapy rehabilitation for people with spinal cord injuries. J Physiother. (2016) 62(1):4–11. 10.1016/j.jphys.2015.11.004 26701156

[3] World Health Organization. International classification of functioning, disability and health. Available at: https://www.who.int/standards/classifications/international-classification-of-functioning-disability-and-health (cited August 15, 2022).

[4] Unger J Singh H Mansfield A Hitzig SL Lenton E Musselman KE. The experiences of physical rehabilitation in individuals with spinal cord injuries: a qualitative thematic synthesis. Disabil Rehabil. (2019 Jun) 41(12):1367–83. 10.1080/09638288.2018.1425745 29334811

[5] Craig A Perry KN Guest R Tran Y Middleton J. Adjustment following chronic spinal cord injury: determining factors that contribute to social participation. Br J Health Psychol. (2015 Nov) 20(4):807–23. 10.1111/bjhp.12143 26037456

[6] Backus D Gassaway J Smout RJ Hsieh CH Heinemann AW DeJong G Relation between inpatient and postdischarge services and outcomes 1 year postinjury in people with traumatic spinal cord injury. Arch Phys Med Rehabil. (2013 Apr) 94(4 Suppl):S165–74. 10.1016/j.apmr.2013.01.012 23527772

[7] Burns AS Santos A Cheng CL Chan E Fallah N Atkins D Understanding length of stay after spinal cord injury: insights and limitations from the access to care and timing project. J Neurotrauma. (2017 Oct) 34(20):2910–6. 10.1089/neu.2016.4935 28245734 PMC5653133

[8] Government of Canada. Seniors’ Falls – An emerging public health crisis (Modified January 14, 2015). Available at: https://www.veterans.gc.ca/eng/resources/health/promotion/fallsp/senfalls (cited August 15, 2022).

[9] Musselman KE Singh H Unger J. Shaping rehabilitation after spinal cord injury: the impact of qualitative research. In: Muller D Hayre C, editors. Enhancing healthcare and rehabilitation: The impact of qualitative research. 1st ed. Boca Raton, FL: CRC Press (2019). p. 141–66.

